# MFE-YOLO: A Multi-Scale Feature Enhanced Network for PCB Defect Detection with Cross-Group Attention and FIoU Loss

**DOI:** 10.3390/e28020174

**Published:** 2026-02-02

**Authors:** Ruohai Di, Hao Fan, Hanxiao Feng, Zhigang Lv, Lei Shu, Rui Xie, Ruoyu Qian

**Affiliations:** 1 School of Cross-Innovation, Xi’an Technological University, Xi’an 710021, China; diruohai@xatu.edu.cn (R.D.);; 2 School of Electronic Information Engineering, Xi’an Technological University, Xi’an 710021, China; 3 School of Aerospace, Xi’an Jiaotong University, Xi’an 710049, China

**Keywords:** Printed Circuit Board (PCB), defect detection, YOLOv5, Bayesian deep learning, uncertainty quantification, attention mechanism, FIoU loss

## Abstract

The detection of defects in Printed Circuit Boards (PCBs) is a critical yet challenging task in industrial quality control, characterized by the prevalence of small targets and complex backgrounds. While deep learning models like YOLOv5 have shown promise, they often lack the ability to quantify predictive uncertainty, leading to overconfident errors in challenging scenarios—a major source of false alarms and reduced reliability in automated manufacturing inspection lines. From a Bayesian perspective, this overconfidence signifies a failure in probabilistic calibration, which is crucial for trustworthy automated inspection. To address this, we propose MFE-YOLO, a Bayesian-enhanced detection framework built upon YOLOv5 that systematically integrates uncertainty-aware mechanisms to improve both accuracy and operational reliability in real-world settings. First, we construct a multi-background PCB defect dataset with diverse substrate colors and shapes, enhancing the model’s ability to generalize beyond the single-background bias of existing data. Second, we integrate the Convolutional Block Attention Module (CBAM), reinterpreted through a Bayesian lens as a feature-wise uncertainty weighting mechanism, to suppress background interference and amplify salient defect features. Third, we propose a novel FIoU loss function, redesigned within a probabilistic framework to improve bounding box regression accuracy and implicitly capture localization uncertainty, particularly for small defects. Extensive experiments demonstrate that MFE-YOLO achieves state-of-the-art performance, with mAP@0.5 and mAP@0.5:0.95 values of 93.9% and 59.6%, respectively, outperforming existing detectors, including YOLOv8 and EfficientDet. More importantly, the proposed framework yields better-calibrated confidence scores, significantly reducing false alarms and enabling more reliable human-in-the-loop verification. This work provides a deployable, uncertainty-aware solution for high-throughput PCB inspection, advancing toward trustworthy and efficient quality control in modern manufacturing environments.

## 1. Introduction

The relentless miniaturization and increasing complexity of electronic devices have placed unprecedented demands on the quality and reliability of Printed Circuit Boards (PCBs). As the foundational substrate for interconnecting electronic components, any defect in a PCB—such as open circuits, shorts, spurs, or mouse bites—can lead to catastrophic system failures [1]. Traditional inspection methods, including manual visual inspection and electrical testing, are not only labor-intensive and costly but also prone to human fatigue and subjective error, making them unsuitable for modern high-volume, precision manufacturing [2,3].

The advent of deep learning, particularly convolutional neural networks (CNNs), has revolutionized automated visual inspection. Among these, single-stage object detectors like the YOLO (You Only Look Once) series have gained prominence for their compelling trade-off between speed and accuracy [4]. The YOLOv5 architecture, in particular, has been widely adopted for industrial tasks, including PCB defect detection [5,6]. However, a significant limitation of standard CNNs, including YOLOv5, is their deterministic nature. They produce point estimates for bounding boxes and class probabilities without any inherent measure of the model’s *confidence* or *uncertainty* in its predictions [7]. In the context of PCB inspection, this translates to a critical shortcoming: the model may output a high-confidence score for a blatant false positive or miss a subtle defect with no indication of its uncertainty. This lack of self-awareness limits the trustworthiness of such systems in real-world, safety-critical applications.

Bayesian deep learning offers a principled framework to address this very issue [7,8]. By treating the weights of a neural network as probability distributions rather than fixed values, Bayesian Neural Networks (BNNs) can naturally quantify *epistemic uncertainty* (model uncertainty due to lack of training data) and *aleatoric uncertainty* (inherent data noise) [9]. This allows for more reliable and interpretable predictions. While full BNNs can be computationally prohibitive, numerous approximate inference techniques, such as Monte Carlo Dropout [8] and ensemble methods [10], have made Bayesian principles practically applicable to large-scale vision tasks. A Bayesian perspective encourages models that are not just accurate but also *well-calibrated*, meaning a prediction with a confidence score of 90% should be correct 90% of the time [11].

Surprisingly, the integration of Bayesian thinking into PCB defect detection remains largely unexplored. Most existing works focus on architectural modifications to boost mean Average Precision (mAP) but neglect the crucial aspect of predictive uncertainty [5,6,12]. This gap is especially pronounced for small defect detection, where features are scarce and ambiguity is high—a scenario where uncertainty estimation is most valuable.

While the aforementioned advancements in deep learning have significantly improved the accuracy of PCB defect detection, the integration of Bayesian principles offers a promising direction to further enhance model reliability and interpretability in real-world applications.

In this paper, we argue that a Bayesian reinterpretation of the PCB defect detection pipeline can yield significant improvements in both performance and reliability. We propose MFE-YOLO, an enhanced YOLOv5 model that embeds Bayesian principles at multiple stages. Our contributions are not merely heuristic improvements but are motivated by a probabilistic understanding of the detection problem. The small size and often ambiguous appearance of PCB defects can be viewed as a source of aleatoric uncertainty, while the limited diversity of public datasets contributes to epistemic uncertainty. Our approach directly tackles these challenges.

First, to mitigate the data limitation and bias present in existing single-background datasets, we construct a multi-background PCB defect dataset encompassing a variety of substrate colors and board shapes. This improves the model’s robustness and generalization across different visual conditions encountered in practice.

Second, we enhance the feature representation by integrating the Convolutional Block Attention Module (CBAM) [13]. From a Bayesian standpoint, the channel and spatial attention mechanisms in CBAM can be interpreted as a form of *feature-wise uncertainty weighting*. By learning to attenuate less informative features (e.g., complex backgrounds) and amplify salient ones (e.g., defect edges), the network is effectively performing a Bayesian marginalization over feature maps, reducing the influence of noisy or confounding inputs. This leads to a more robust and focused feature extraction process.

Third, we identify a key weakness in the commonly used CIoU loss function for bounding box regression. Its reliance on aspect ratio difference can fail when the predicted and target boxes have identical ratios but different scales. To address this, we redesign the loss function, proposing FIoU. More importantly, we frame this redesign within a probabilistic context. The FIoU loss incorporates absolute dimensional differences, which can be linked to modeling the uncertainty in the scale of the bounding box. This results in a more stable and accurate regression, particularly for small objects where localization error is most critical.

**In summary, the main contributions of this paper are as follows:**

(1) Construction of a multi-background color PCB dataset, serving as a broader prior distribution for training a robust, uncertainty-aware defect detector.
(2) Proposal of an improved YOLOv5-based PCB defect detection model, where the integrated CBAM attention mechanism acts as a Bayesian feature weighting module to suppress background noise and enhance defect-specific features.
(3) Redesign of the FIoU loss function based on the CIoU loss, incorporating a probabilistic perspective on bounding box scale to improve regression accuracy and implicitly model localization uncertainty.

Through extensive ablation studies and comparisons, we demonstrate that our Bayesian-enhanced MFE-YOLO not only achieves superior detection accuracy but also paves the way for more reliable, calibrated, and trustworthy automated optical inspection systems.

## 2. Basic Principles

### 2.1. Definition of Defects

Currently, numerous standards for PCB defect detection have been established domestically and internationally, with different standards focusing on slightly varying aspects. Among them, the most widely applied and authoritative is the IPC standard (Institute of Printed Circuits), developed by the global association for the electronic circuits and electronic interconnections industry. The IPC standard specifies corresponding acceptance criteria for each production stage of PCBs. The latest IPC standard regarding the acceptance conditions for PCB surface appearance is IPC-A-600K [14]. This standard classifies PCB surface defects, and Table 1 presents the classification of surface defect types according to IPC-A-600K. From this, six types of defects—Open Circuit, Short, Spur, Mouse Bite, Spurious Copper, and Missing Hole—have been selected as the focus of this study.

In accordance with the IPC-A-600K standard, this study focuses on the detection of six critical and commonly encountered surface defects in PCBs, which are visually defined as follows:

(1) Open Circuit: A break or discontinuity in a conductive trace, resulting in an incomplete electrical path. Visually, it appears as a gap or separation in the copper line.
(2) Short (Short Circuit): An unintended electrical connection between two or more separate conductive elements, often appearing as a thin, erroneous bridge of copper between adjacent traces or pads.
(3) Spur: An undesired, small protrusion of copper extending from the edge of a trace or pad, resembling a tiny spike or branch.
(4) Mouse Bite: An indentation or notch along the edge of a trace or copper area, giving the appearance of having been “bitten” out, often resulting from incomplete etching. It should be noted that within the IPC-A-600K classification, this specific etching defect is listed under “Other defect types” and is synonymous with the term “Notch” in the same table, as shown in Table 1. This distinguishes it from the panel design feature also commonly called “mouse bites”, which are intentional perforations for separating boards from a manufacturing panel.
(5) Spurious Copper: Unwanted residual copper material left on the substrate in areas where it should have been completely etched away, appearing as isolated or connected copper flecks.
(6) Missing Hole: The absence of a drilled or punched hole in a designated pad or via location, where a hole should be present according to the design.

These six defect types encompass a range of geometric and photometric characteristics, from small, localized anomalies (e.g., Spurs, Mouse Bites) to larger, more diffuse features (e.g., Spurious Copper). The majority, particularly Spurs, Mouse Bites, and small Shorts/Open Circuits, fall into the small target category as per the SPIE definition (occupying <0.12% of the image area), presenting significant challenges for accurate detection and localization against complex PCB backgrounds.

### 2.2. PCB Analysis of Difficulties in Defect Detection

YOLOv5 is the most widely used single-stage object detection algorithm in the field of target detection. It performs differently when detecting targets of varying scales, showing good detection results for normal, medium, and large-sized targets. However, it often misses small targets during detection. Through a comprehensive analysis of the YOLOv5 network model structure, its poor performance in detecting PCB defects is mainly due to the following three reasons:

(1) The complex background and diverse surface colors cause the background to resemble target defects, affecting detection results;
(2) An excessively large receptive field results in defects occupying few features in the feature map, low precision in bounding box regression, and the inclusion of numerous surrounding area features, thereby impacting detection results;
(3) The defect target pixels are too small relative to the entire image, and the model’s excessive downsampling results in the output target occupying only one or two pixels.

### 2.3. YOLOv5 Defect Detection Model

**Figure 1:**
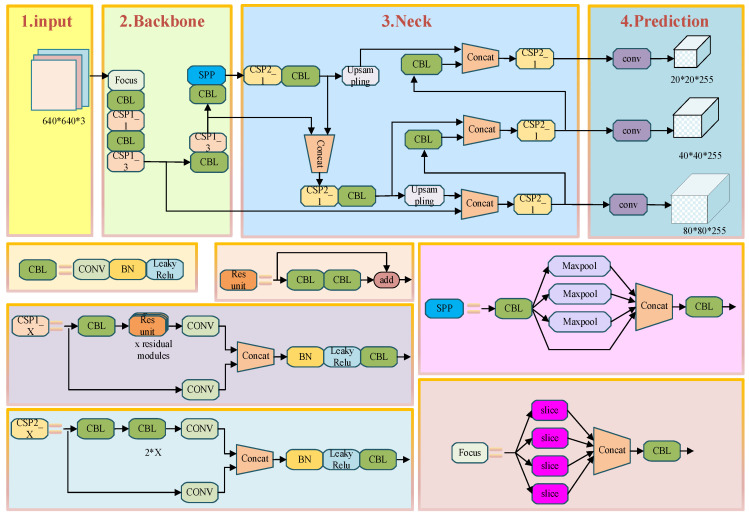
YOLOv5 network model diagram.

The network structure of YOLOv5 mainly consists of three parts: Backbone, Neck, and Head. The Backbone, as the feature extraction module, is primarily composed of focus, CBL, CSP, SPP and four other modules, whose main function is to extract feature information from the image. The Neck, as the key part connecting the preceding and following sections of the entire network, is mainly used to fuse the feature information extracted by the Backbone, generating a feature pyramid. The feature pyramid enables the model to learn features at different scales, improving its ability to detect objects of varying sizes and thereby enhancing the overall detection performance of the network. The Head part, as the prediction section of the entire network, primarily utilizes the multi-scale feature information output by the Neck to predict the location and category of targets from these output features. The YOLOv5 network model is shown in Figure 1.

#### 2.3.1. Backbone Feature Extraction

In YOLOv5, the Backbone, as the feature extraction module, is mainly composed of Focus, CBL, CSP, and SPP modules: ① The Focus module was first proposed by the YOLOv5 authors. It primarily utilizes a near 2× downsampling operation to extract pixel values from the image at intervals. This process expands a single image into four independent feature layers without losing original information. These four feature layers are then stacked sequentially, converting width and height dimensional information into channel dimensions. As a result, both the width and height of the image are reduced to half. Although the Focus module does not improve the model’s mAP, it effectively reduces computational load by decreasing the number of parameters while preserving information, thereby enhancing the overall inference speed of the model. ② The CBL module consists mainly of a convolution layer (Conv), batch normalization (BN), and a ReLU activation function. As an independent module, CBL exclusively uses 3 × 3 convolutions and is primarily responsible for image downsampling. Each time an image passes through a CBL module, its spatial dimensions are reduced by half. ③ The CSP (Cross-Stage Partial Network) module reduces computational load by splitting the input feature map into two parts for convolutional operations. It then merges the results from these two cross-stage convolutional branches, enriching the gradient flow within the network. The authors of YOLOv5 designed two distinct CSP structures for the Backbone and Neck sections, respectively, based on the original CSP architecture. This was later improved into the C3 module. ④ The SPP (Spatial Pyramid Pooling) module, also known as the spatial pyramid pooling structure, was inspired by the SPPNet [15] proposed by Kaiming He in 2015. Regardless of the input image size, the SPP structure can produce fixed-size outputs. It converts input images of varying sizes into pooled features of the same scale by using multiple pooling kernels of different sizes. The resulting feature maps of different scales are then concatenated. This not only expands the receptive field but also integrates multi-scale features, effectively enhancing the network’s ability to extract feature information.

#### 2.3.2. Neck Feature Extraction and Fusion

**Figure 2:**
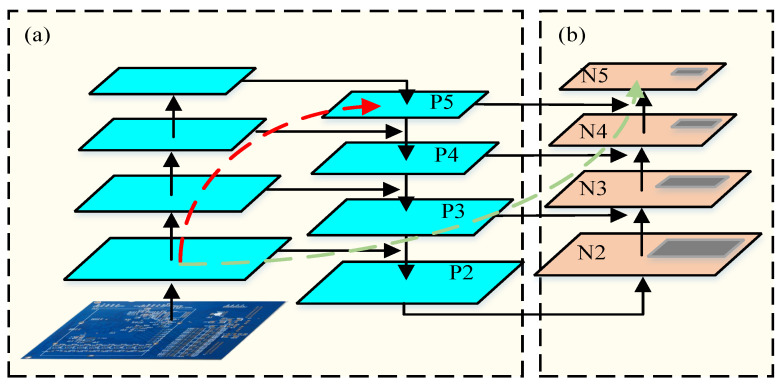
PANANet Network Structure. (**a**) FPN Backbone. (**b**) Bottom-up path augmentation.

The Neck section of YOLOv5 is primarily composed of FPN [16] and PANet [17] structures. Its main function is to integrate feature information extracted from various convolutional layers to generate a feature pyramid, thereby enhancing the model’s capability to detect objects at different scales and improving its adaptability to targets of varying sizes. The FPN (Feature Pyramid Network) structure is designed to integrate high-level semantic features with low-level detailed information from the network, effectively improving the detection performance for objects of different sizes. In contrast, PANet (Path Aggregation Network) builds upon FPN by incorporating an upsampling operation after the downsampling process—enabling bottom-up information fusion. This effectively allows higher layers to integrate more detailed positional information, further boosting detection performance across different object scales. The network structure of PANet is illustrated in Figure 2.

#### 2.3.3. Head Prediction Regression

**Figure 3:**
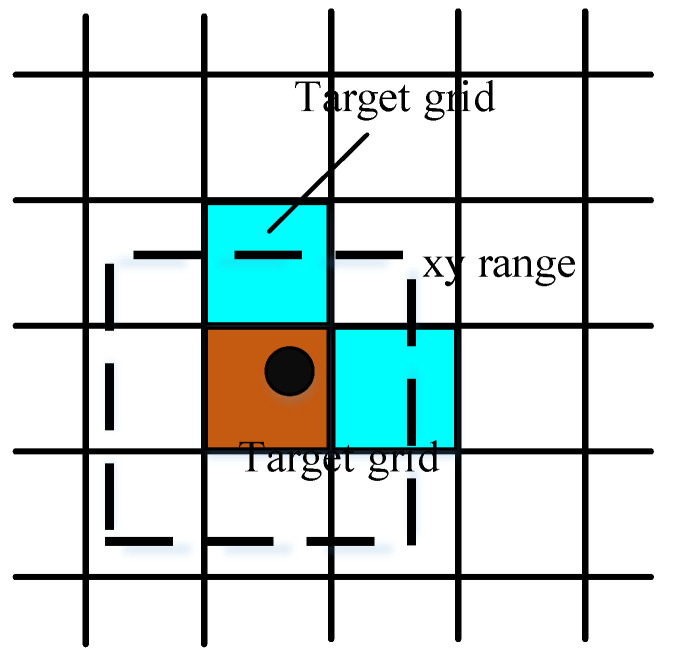
YOLOv5 Positive Sample Selection Illustration.

The authors of YOLOv5 designed three Detect detectors in the Head section, which utilize Anchors of different scales, sizes, and positions as fixed reference boxes to perform predictions on three differently scaled feature maps output from the Neck. As shown in the network architecture in Figure 1, YOLOv5 takes an input image size of 3 × 640 × 640 and outputs feature maps of three different scales: 80 × 80, 40 × 40, and 20 × 20. YOLOv5 primarily uses predefined Anchors to predict ground truth bounding boxes. By adopting a cross-grid matching strategy, it effectively increases the number of positive Anchors at each layer. The method first calculates the width-to-height ratios between candidate bounding boxes and the Anchors across the three feature layers. Based on a predefined threshold, bounding boxes with insufficient matching scores are discarded and considered as background in that layer. Among the remaining bounding boxes, those with the highest matching scores are selected. The algorithm then identifies the grid cell in which the ground truth bounding box falls and locates the two nearest neighboring cells according to the actual matching degree. These adjacent cells are also designated as responsible for predicting the same bounding box. A schematic diagram of YOLOv5’s positive sample selection is shown in Figure 3. In the figure, the black dot represents the center point of the ground truth object, while the blue dots indicate the center points of additional positive samples identified based on matching criteria.

## 3. The Method in This Paper

**Figure 4:**
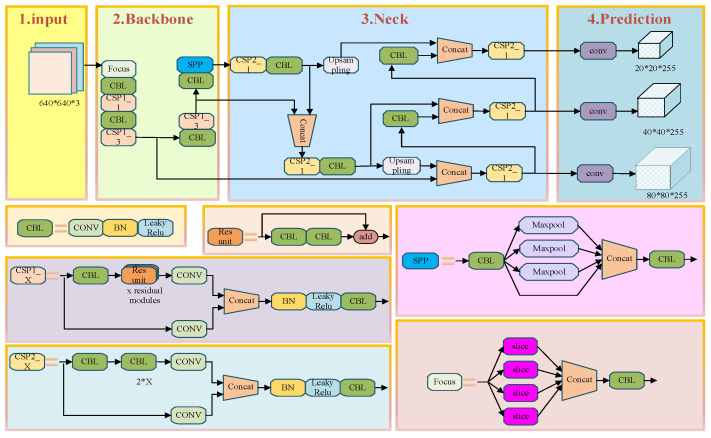
YOLOv5 Positive Sample Selection Diagram.

The PCB defect detection algorithm studied in this paper primarily focuses on identifying surface defects on PCBs. According to the definition provided by the International Society for Optics and Photonics (SPIE), a target occupying less than 0.12% of the entire image is defined as a small target. Based on this criterion, most of the targets examined in this study fall into the small target category. Therefore, the selected network must not only ensure fast, real-time, and accurate detection but also—and especially—maintain a high detection accuracy for small targets. Considering the challenges in PCB defect detection, it is noted that PCBs used in different applications may vary in trace width, layout, and background color. This often leads to complex background information and limited target feature representation. To address these issues, this paper proposes an improved YOLOv5 object detection algorithm for detecting six types of defects in PCBs. The improvements mainly focus on three components: the Backbone, the loss function, and the Head. The architecture of the modified YOLOv5 model is shown in Figure 4.

### 3.1. Improvement of the Backbone

**Figure 5:**
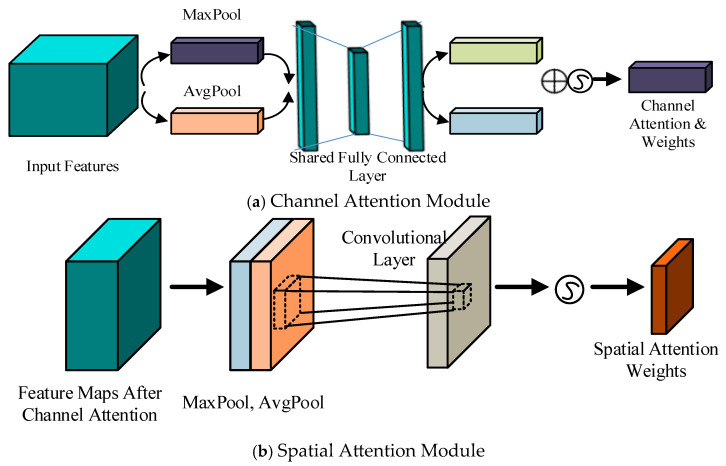

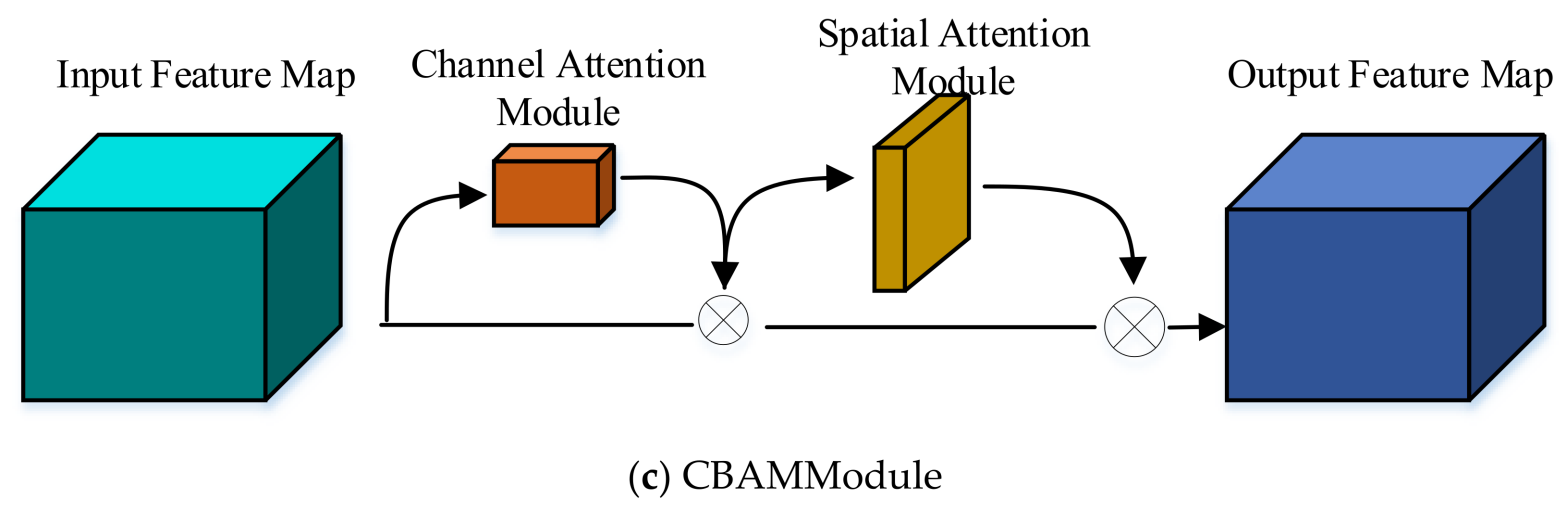
CBAM Structure Diagram.

In YOLOv5, the Backbone, which serves as the feature extraction module, is primarily composed of four key components: the Focus, CBL, CSP, and SPP modules. Research has shown that introducing an attention mechanism into the original network can enhance the model’s ability to focus on locally salient information, thereby effectively improving the accuracy of object detection. To leverage this, the present study incorporates the CBAM (Convolutional Block Attention Module) into the original Backbone structure. CBAM is an attention mechanism that combines both spatial and channel attentions. This module sequentially infers attention weights along the channel and spatial dimensions from the input feature map. The original input features are then multiplied by the resulting attention weights to achieve adaptive feature refinement. The structure of the CBAM is illustrated in Figure 5.

Among them, the feature map undergoes channel and spatial attention mechanisms, sequentially reassigning weight coefficients to different regions in the channel and spatial dimensions. This reduces the weights of non-target regions and weakens the features of background areas while further enhancing the weights of target regions and highlighting target features. The channel attention expression is:

(1) $$M_{S}(F)=\sigma \left(f^{7\times 7}([AvgPool(F);MaxPool(F)])\right)\;=\left[0,1\right]\sigma \left(f^{7\times 7}\left(\left[F_{avg}^{s};F_{max}^{s}\right]\right)\right)$$

where σ is the sigmoid operation,${\;W}_{0}\in R^{C/r\times C}$, C is the number of neurons, r is the reduction ratio, $W_{1}$ are the weights of the multilayer perceptron, $F_{avg}^{c}$ represents average pooling, and $F_{max}^{c}$ represents max pooling.

The spatial attention expression is:

(2) $$M_{S}(F)=\sigma \left(f^{7\times 7}([AvgPool(F);MaxPool(F)])\right)\;=\left[0,1\right]\sigma \left(f^{7\times 7}\left(\left[F_{avg}^{s};F_{max}^{s}\right]\right)\right)$$

where 7×7 represents the size of the convolution kernel, $F_{avg}^{c}$ represents average pooling, and $F_{max}^{c}$ represents max pooling.

**Figure 6:**
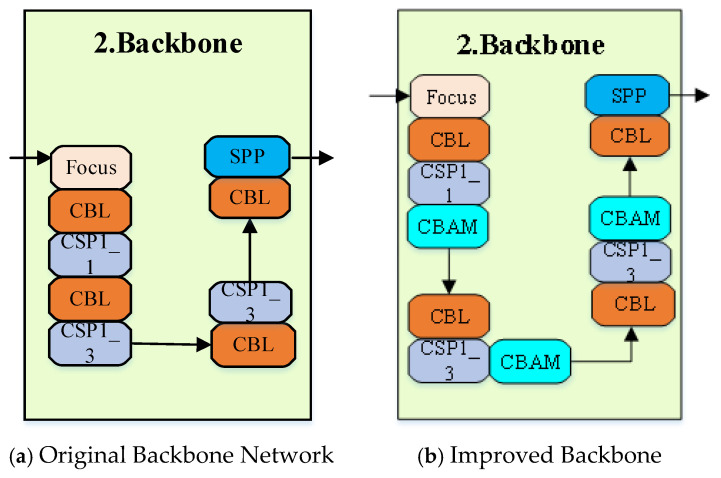
Improved Backbone.

The improvement in this paper primarily involves adding a CBAM layer after the CSP layer in the Backbone network. After the feature map passes through the CBAM layer, it is reweighted based on the learned attention coefficients, enabling the network to focus more on critical regions and effectively enhancing the feature representation capability of the model. The modified Backbone network is illustrated in Figure 6.

### 3.2. Neck and Redesign of the Head Part

An analysis of the six types of PCB defects detected in this study reveals that the vast majority of these defects occupy less than 0.1% of the total PCB area, categorizing them as small targets. However, the original YOLOv5 model exhibits a significantly higher missed detection rate for small targets compared to larger ones. The primary reason for this limitation lies in the design of the YOLOv5 network. The Head section of the original model contains only three detection layers. Given an input image size of 640 × 640, the output feature maps have dimensions of 80 × 80, 40 × 40, and 20 × 20, respectively. These correspond to the detection of targets larger than 8 × 8, 16 × 16, and 32 × 32 pixels. In the actual PCB images collected for this system, after resizing to 640 × 640, the defects are also scaled down. As a result, many of the actual defect targets become smaller than the minimum detectable size supported by the original detection layers, leading to relatively low precision in defect detection.

**Figure 7:**
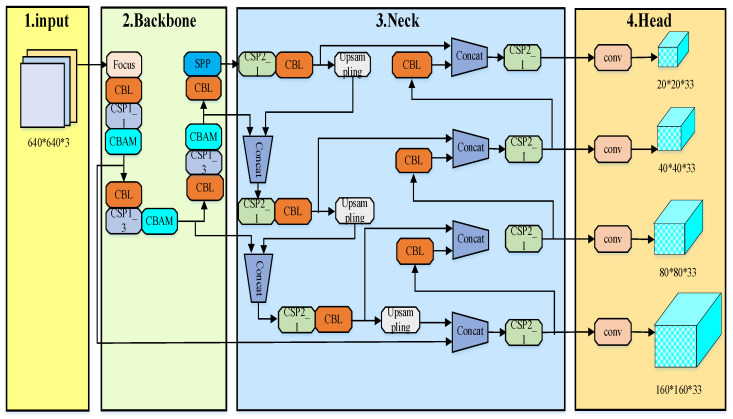
Improved Head Network Diagram.

Based on the aforementioned analysis of the detection targets and the YOLOv5 network model, this study draws inspiration from the improvement concept of TPH-YOLOv5 [18] and enhances the original Head component of YOLOv5 by adding a dedicated detection head specifically designed for small target defect detection. As a result, the network now outputs feature maps at four different scales: 160 × 160, 80 × 80, 40 × 40, and 20 × 20. Compared to the original network, which could detect targets as small as 8 × 8 pixels, the improved model is capable of detecting targets as small as 4 × 4 pixels, significantly enhancing the network’s sensitivity to small targets. Although the addition of a small target detection head increases the computational load and complexity of the model to some extent, it substantially improves the detection performance for small targets and effectively enhances the accuracy of small target detection. The structure of the modified Head network is illustrated in Figure 7.

### 3.3. Loss Function Redesign

The loss function in object detection tasks typically consists of classification loss and bounding box regression loss. This section focuses on improving the bounding box regression loss. Currently, commonly used bounding box regression losses in object detection are mostly improved based on IoU (Intersection over Union) loss. The formula for calculating IoU is as follows:

(3) $$IoU=\frac{\left|A\cap B\right|}{\left|A\cup B\right|}$$

where A is the ground truth box area, and B is the predicted box area.

IoU loss can measure the distance between predicted bounding boxes and ground truth boxes, thereby accurately reflecting detection performance. However, as can be seen from the calculation method above, when the predicted box and the ground truth box do not intersect, the IoU value becomes zero. In such cases, IoU loss fails to effectively quantify the distance between the two boxes, which hinders accurate learning of bounding box regression.

The authors of YOLOv5 adopt CIoU (Complete Intersection over Union) as the bounding box regression loss function. CIoU takes into account the distance between the target and the anchor, the overlap rate, and the scale, which makes the bounding box regression more stable and avoids the divergence issue that occurs during training with standard IoU. The formula for calculating CIoU is as follows:

(4) $$CIoU=IoU-\frac{\rho^{2}\left(b,b^{gt}\right)}{c^{2}}-\alpha v$$

where b are the coordinates of the center point of the predicted box, $b^{gt}$ are the coordinates of the center point of the ground truth box, $\rho^{2}\left(b,b^{gt}\right)$ represents the Euclidean distance between the center points of the predicted box and the ground truth box, and c represents the diagonal distance of the smallest enclosing box that contains both the predicted box and the ground truth box, α and v. The calculation formulas are:

(5) $$v=\frac{4}{\pi^{2}}{\left(\arctan\frac{w^{gt}}{h^{gt}}-\arctan\frac{w}{h}\right)}^{2}$$

(6) $$\alpha =\frac{v}{1-IoU+v}$$

where $h^{gt}$ and $w^{gt}$ represent the width and height of the ground truth box, and h and w represent the width and height of the predicted box.

An analysis of CIoU reveals that it employs the relative ratios of width and height rather than their actual values. According to the definition of v, whenever the predicted bounding box satisfies the condition $\left\{(w=kw^{gt},h=kh^{gt})|ke\in R^{+}\right\}$, the penalty term based on relative proportions in CIoU ceases to function. To address this limitation, this paper proposes a redesigned loss function named FIoU, building upon the concept of CIoU. While the original CIoU loss uses the Euclidean distance between the centers of the predicted and ground truth bounding boxes as a penalty term, the proposed FIoU loss adopts a simplified single distance metric between the centers, reducing computational complexity compared to the Euclidean distance. Furthermore, to overcome the drawback of CIoU’s reliance on relative width and height ratios rather than actual values, FIoU incorporates the absolute differences in width and height between the predicted and ground truth boxes as penalty terms. This redesign effectively resolves the limitations associated with the use of relative proportions in CIoU. The formula for the proposed FIoU loss is defined as follows:

(7) $$FIOU=IoU-(\frac{center\_x}{Cw}+\frac{center\_y}{Ch}+\frac{\mathrm{side}\_w^{2}}{Cw^{2}}+\frac{side\_h^{2}}{Ch^{2}})$$

where Cw and Ch represent the width and height of the smallest enclosing box that contains both the predicted box and the ground truth box, center_x and center_y represent the distance between the center points of the ground truth box and the predicted box, and side_w and side_h represent the differences in width and height between the ground truth box and the predicted box. The center_x, center_y, center_x, and center_y calculation formulas are:

(8) $$\left\{\begin{array}{c} center\_x=\left|center\_gx-center\_px\right|\\ center\_y=\left|center\_gy-center\_py\right| \end{array}\right.$$

(9) $$\left\{\begin{array}{c} side\_h=\left|g\_h-p\_w\right|\\ side\_w=\left|g\_w-p\_w\right| \end{array}\right.$$

where center_gx and center_gy represent the coordinates of the center point of the ground truth box; center_px and center_py represent the coordinates of the center point of the predicted bounding box; g_w and g_h represent the width and height of the ground truth bounding box; and p_w and p_h represent the width and height of the predicted bounding box.

The proposed FIoU shares the common goal of enhancing bounding box regression with several recent IoU variants, yet it is designed with distinct motivations and formulations tailored for small defect detection. EIoU (Efficient IoU) [19] decomposes the geometric loss into center distance, width, and height differences using the normalized squared distances $(\Delta w/C_{w}{)}^{2}+(\Delta h/C_{h}{)}^{2}$. While effective, its width/height penalty remains a relative measure scaled by the enclosing box dimensions $\left(C_{w},C_{h}\right)$. In contrast, FIoU directly penalizes the absolute squared differences $\frac{\Delta w^{2}}{C_{w}^{2}}+\frac{\Delta h^{2}}{C_{h}^{2}}$, which provides a more direct and sensitive penalty for scale errors of small objects, where absolute pixel deviations are critical. SIoU (Scylla IoU) [20] introduces an angular cost term to reshape the regression trajectory, prioritizing directionality. FIoU [21], however, maintains a focus on the fundamental geometric factors (center, width, height) without introducing angular complexity, ensuring stable and interpretable gradients, especially beneficial when dealing with the often near-axis-aligned PCB defects. From a Bayesian perspective, the explicit penalty on absolute width/height differences in FIoU can be interpreted as imposing a prior on the scale uncertainty of the bounding box. This formulation encourages the model to not only match the aspect ratio but also to be confident in the absolute size of the predicted region, thereby providing an implicit measure of localization uncertainty related to object scale—a crucial aspect for reliably assessing small, ambiguous defects.

In summary, the formula for the redesigned bounding box regression loss function, FIoU loss, proposed in this paper, is as follows:

(10) $$FIOU_{loss}=1-IoU+\left(\frac{center_{x}}{Cw}+\frac{center_{y}}{Ch}+\frac{side_{w}^{2}}{Cw^{2}}+\frac{side_{h}^{2}}{Ch^{2}}\right)$$

## 4. Experimental Testing and Result Analysis

The experiments for the defect detection algorithm in this study were conducted on a Windows 10 operating system, where a model based on the improved YOLOv5 defect detection algorithm was implemented and trained. The development and testing were carried out on a computer platform equipped with an AMD 5800X processor (Santa Clara, CA, USA), an NVIDIA Quadro M6000 graphics card, and 32 GB of RAM (Santa Clara, CA, USA). The deep learning environment utilized PyTorch 1.7.1, Python 3.8, and CUDA 11.3. To select the best-performing network model, the dataset was divided into training, validation, and test sets in an 8:1:1 ratio. The resulting sample sizes for the three subsets were 15,796 images, 1975 images, and 1975 images, respectively. The training parameter settings for the network model are provided in Table 2.

### 4.1. Construction of the Dataset

The construction of a defect dataset primarily serves the purpose of supporting the defect detection network model. To address the issue of insufficient defect samples, this study enhances the original public dataset by incorporating manually annotated images, thereby increasing the diversity of the dataset. The PCB defect dataset constructed in this work consists of two main sources: (a) public datasets and (b) images acquired using digital imaging devices. Since the PCBs in public datasets typically have a uniform green background, they cannot meet the requirements for detecting defects in PCBs with diverse background colors. Therefore, this study combines defective PCB images collected through flatbed scanning to construct a multi-background-color PCB defect dataset. Due to the scarcity of defective PCBs in practice, the currently collected defect samples are insufficient to meet the demands of detection accuracy and model generalization. To further expand the number of defect samples, this paper employs data augmentation techniques to increase the quantity of defective samples, thereby improving the robustness and generalization ability of the defect detection model.

**Figure 8:**
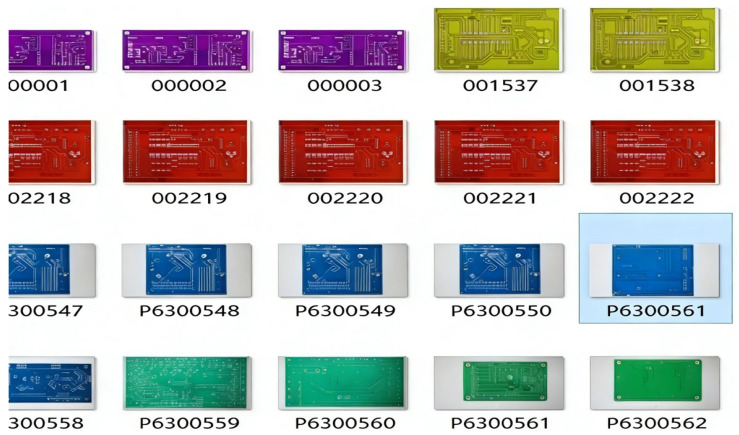
Examples of Some Dataset Images.

The constructed multi-background PCB dataset encompasses a wide variety of PCB appearances to ensure robustness and generalizability. The PCBs vary in shape, including rectangular, circular, and irregular layouts. The background colors extend beyond the conventional green to include blue, red, black, and white substrates, simulating real-world manufacturing scenarios. This diversity in shape and appearance helps the model learn invariant features across varying PCB designs, thereby strengthening its applicability in practical industrial inspection systems. Some examples of the dataset are shown in Figure 8.

Based on the IPC-A-600K standard, this study establishes a defect detection criterion for printed circuit boards (PCBs). The public PCB dataset used is from the Open Intelligent Robotics Laboratory of Peking University, which consists of 1386 images—including 693 original images and another 693 generated by randomly rotating the original images. The construction of a defect dataset aims to support the training of defect detection network models. To address the issue of insufficient defective samples and the limitation of the public dataset (which only contains PCBs with a uniform green background, thus failing to meet the need for detecting defects in PCBs with diverse background colors), this study augments the original public dataset by adding defect samples with varied background colors to enhance sample diversity. A self-built dataset of 709 images was created, resulting in a combined dataset of 1402 images. To further expand the dataset, common data augmentation techniques were applied, including random flipping, random rotation, random brightness adjustment, and random noise injection. After augmentation, the total number of images reached 5120. To adapt the images to the network and minimize information loss during resizing, all augmented images were cropped to a unified size of 1000 × 1000 pixels. The final defect dataset contains 19,746 images. Table 3 provides a breakdown of the quantity of each type of defect after data augmentation.

### 4.2. Evaluation Metrics

To objectively evaluate the effectiveness of the improvements in both the original and modified network models, this study employs mean Average Precision (mAP) as the key evaluation metric, which represents the mean of Average Precision (AP) across all categories. The calculation of detection performance is based on a confusion matrix, where True Positives (TP) denote positive samples correctly predicted as positive, False Negatives (FN) indicate positive samples incorrectly predicted as negative, False Positives (FP) refer to negative samples mistakenly predicted as positive, and True Negatives (TN) represent negative samples correctly predicted as negative. AP is defined as the area under the Precision–Recall (PR) curve, with Precision and Recall calculated from the confusion matrix to comprehensively assess the detection accuracy and robustness of the model:

(11) $$\mathrm{precision}=\frac{TP}{TP+FP}$$

(12) $$\mathrm{recall}=\frac{TP}{TP+FN}$$

In accordance with the definitions of mAP in COCO and VOC datasets, the specific mAP metric is further categorized into mAP@0.5 and mAP@0.5:0.95. Here, mAP@0.5 represents the average precision calculated at an IoU threshold of 0.5, while mAP@0.5:0.95 denotes the average of AP values computed over multiple IoU thresholds ranging from 0.5 to 0.95 with a step size of 0.05. A higher mAP value indicates better overall performance of the network model and higher accuracy in defect detection.

### 4.3. CBAM Experiment

**Figure 9:**
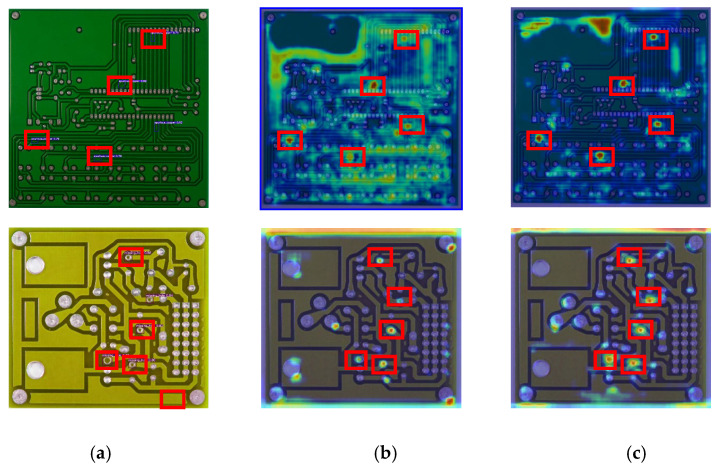
Comparison chart of CBAM experimental results. (**a**) Original Network Heatmap. (**b**) Improved Network Heatmap. (**c**) Improved network heat map.

To validate the effectiveness of incorporating the CBAM attention mechanism, this study employs heatmaps to visualize and compare the feature representations of both the modified network with CBAM and the original network. The improved architecture integrates the attention mechanism at three specific locations. For analysis, the feature heatmaps from the 17th layer of both networks are extracted and superimposed onto the original image through weighted fusion. Figure 9 illustrates a comparative visualization of the original image fused with heatmaps from the 17th layer of both the baseline network and the CBAM-enhanced network. 

As intuitively observed from Figure 9, the incorporation of the CBAM attention mechanism into the original network significantly enhances its ability to capture defect location information and increases the model’s focus on defective regions. This effectively demonstrates that, compared to the baseline model, the CBAM-augmented network exhibits substantially improved attention to local defect features while suppressing interference from global non-defective background information. For defect detection tasks involving small targets and complex backgrounds, the enhanced network can more rapidly locate and emphasize defect-related features, thereby further improving detection accuracy.

While the CBAM enhances feature discrimination, it is crucial to quantify its impact on model complexity and inference efficiency for practical deployment. To this end, we measure and compare the key computational metrics—parameters, FLOPs, and inference frames per second (FPS)—for the baseline YOLOv5s model and its variants integrated with different attention mechanisms (CBAM, SE, CA, ECA). The results are summarized in Table 4. All FPS measurements are conducted on the same NVIDIA Quadro M6000 GPU with a batch size of 1, input size of 640 × 640, and averaged over 100 runs.

To further validate the effectiveness of the CBAM attention mechanism, this study incorporates four different attention mechanisms—CBAM, SE, CA, and ECA—into the original network for training. A comparative analysis of the detection accuracy among these four attention-enhanced variants is conducted based on the experimental results. Table 4 presents the performance metrics of the original network augmented with each of the four attention mechanisms.

As presented in Table 4, incorporating the CBAM introduces a modest increase in computational overhead. Compared to the baseline, CBAM adds approximately 1.0% more parameters and 3.2% more FLOPs, resulting in an inference speed of 158 FPS, which represents a 4.2% decrease relative to the baseline’s 165 FPS. In contrast, other attention mechanisms like SE, CA, and ECA incur negligible or zero parameter growth and less than 1% FLOPs increase, with correspondingly smaller impacts on FPS (0.6% to 1.8% reduction).

However, this marginal computational cost is justified by the significant gain in detection accuracy. CBAM achieves the highest mAP@0.5 (87.5%) and mAP@0.5:0.95 (55.1%) among all attention variants, outperforming the second-best (CA) by 1.0% and 0.7%, respectively. The CBAM-enhanced model maintains a high inference speed well above typical real-time requirements (e.g., >30 FPS for most industrial inspection lines), demonstrating an excellent accuracy-efficiency trade-off. This analysis confirms that while CBAM does increase computational load, the resultant performance improvement for small, ambiguous PCB defects substantiates its inclusion, and the model remains highly suitable for deployment in real-time automated optical inspection systems.

### 4.4. Loss Function Experiment

**Figure 10:**
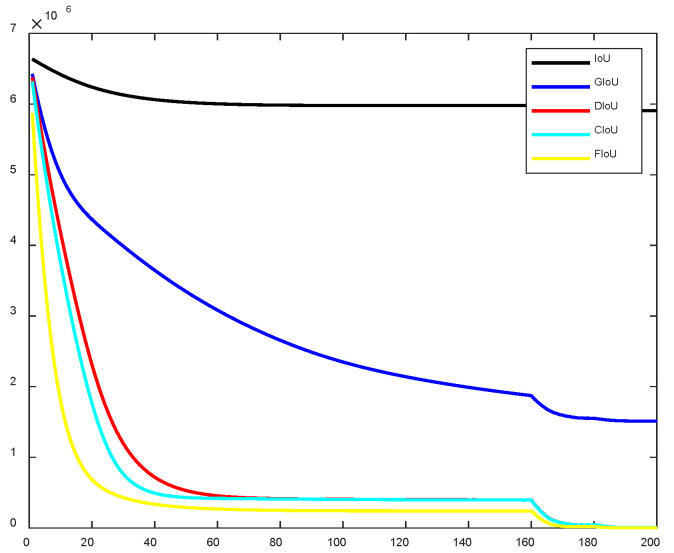
Comparison diagram of various loss curves.

An effective loss function can accelerate network convergence and improve detection accuracy. To validate the effectiveness of the proposed loss function, this study employs the simulation tool introduced in DIoU to compare the regression loss curves during training for IoU, GIoU, DIoU, CIoU, and the proposed FIoU. The comparison of the loss curves corresponding to these five IoU variants is shown in Figure 9. As is visually evident from Figure 10, the proposed FIoU loss exhibits faster convergence speed and lower loss values compared to IoU, GIoU, DIoU, and CIoU.

**Figure 11:**
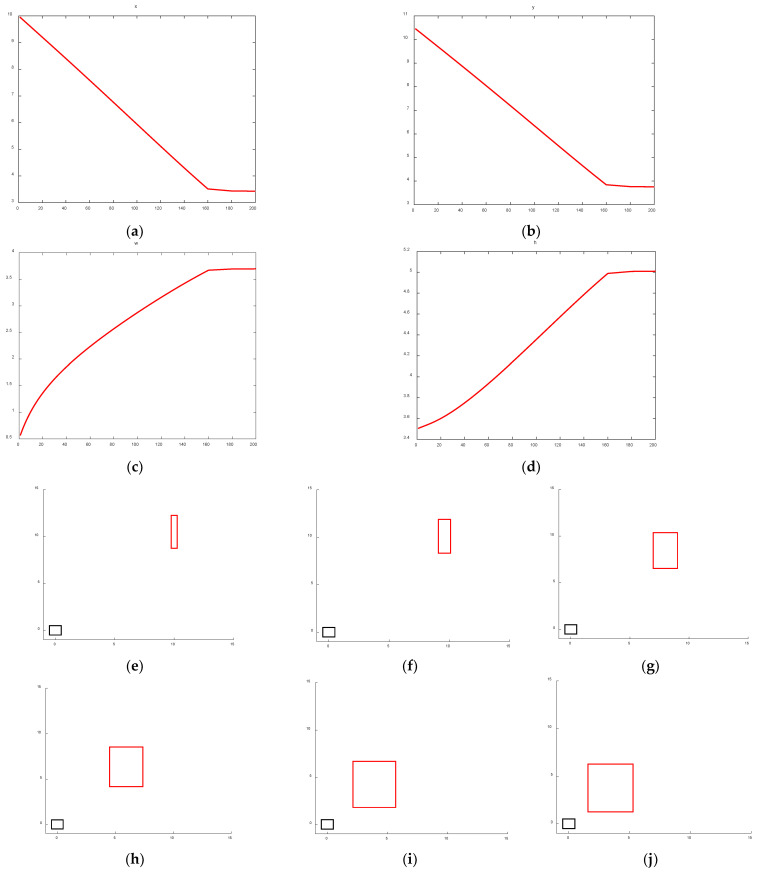
CIoU prediction box regression changes. (**a**) Prediction box center point coordinate X. (**b**) Prediction box center point coordinate Y. (**c**) Prediction box width. (**d**) Prediction box height. (**e**) Initial prediction box and ground truth box positions. (**f**) Trained 10 times. (**g**) Trained 50 times. (**h**) Trained 100 times. (**i**) Trained 150 times. (**j**) Trained 200 times.

To further validate the effectiveness of the proposed FIoU loss function, this study compares the regression behavior of the bounding box center point, width, height, and position between the original CIoU Loss used in YOLOv5 and the improved FIoU loss. Specifically, the ground truth bounding box (gt, shown in black) is set as [0, 0, 1, 1], while the predicted bounding box (pred, shown in red) is defined as [10, 10.5, 0.5, 3.5]. The regression changes in the predicted box’s center point, width, height, and position under CIoU loss are illustrated in Figure 11.

**Figure 12:**
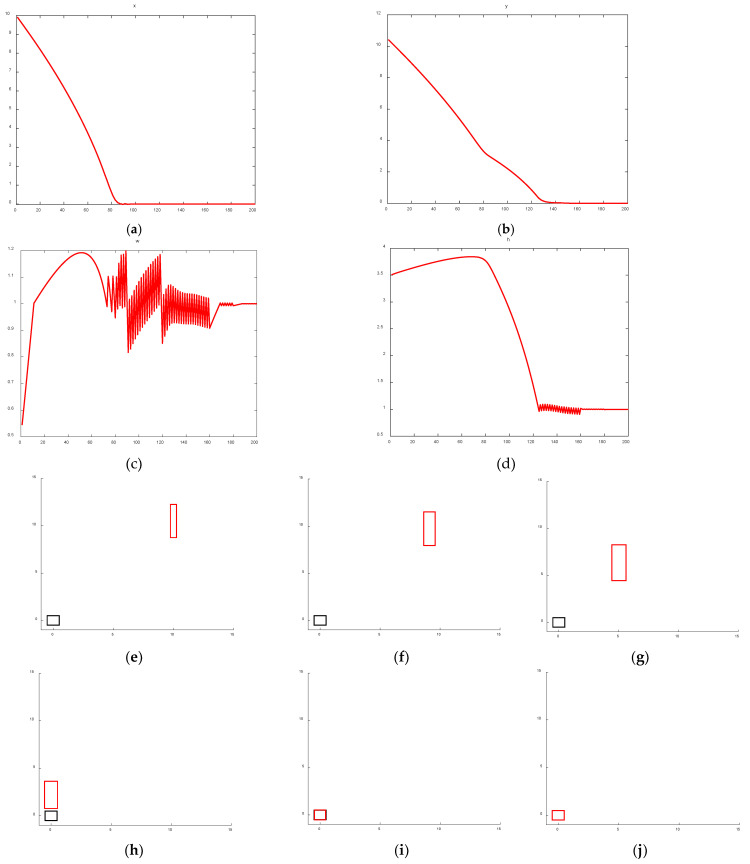
FIoU Prediction box regression changes. (**a**) Prediction box center point coordinate X. (**b**) Prediction box center point coordinate Y. (**c**) Prediction box width. (**d**) Prediction box height. (**e**) Initial prediction box and ground truth box positions. (**f**) Trained 10 times. (**g**) Trained 50 times. (**h**) Trained 100 times. (**i**) Trained 150 times. (**j**) Trained 200 times.

The regression process of the bounding box center point, width, height, and position based on the proposed FIoU loss is illustrated in Figure 12.

An analysis of the bounding box regression behavior—including center point, width, height, and position—between CIoU and the proposed FIoU reveals clear advantages of the latter. As shown in Figure 10 and Figure 11a,b, after 100 training iterations, the distance between the predicted and ground truth center points is significantly larger with CIoU compared to FIoU. Furthermore, Figure 10 and Figure 11c,d indicate that FIoU achieves close alignment with the ground truth width and height after approximately 130 iterations. In contrast, CIoU—which focuses on relative aspect ratio rather than absolute size—converges to the correct width-to-height ratio around 160 iterations but fails to match the actual dimensions of the ground truth box. A comprehensive comparison of Figure 10 and Figure 11e–j further confirms that the proposed FIoU loss enables faster, more accurate, and more stable regression than the original CIoU loss used in YOLOv5, demonstrating the effectiveness of the FIoU design.

To further substantiate the advantages of the proposed FIoU, we extend our comparison to include two other advanced IoU-based loss functions: EIoU and SIoU. All experiments are conducted under the same settings as in Section 4.4. The quantitative results of mAP@0.5 and mAP@0.5:0.95 for YOLOv5s equipped with different regression losses are summarized in Table 5.

The results in Table 5 demonstrate that FIoU achieves the highest detection accuracy among all compared loss functions. While EIoU and SIoU both outperform the original CIoU, showing the benefits of their respective designs (decoupled dimension loss and angular consideration), FIoU surpasses them by a clear margin (+1.4% and +1.1% in mAP@0.5 over EIoU and SIoU, respectively, and +2.5% and +2.2% in mAP@0.5:0.95). This superior performance validates our design rationale: for the specific challenge of small PCB defect detection, directly and sensitively penalizing absolute scale mismatches (as in FIoU) is more effective than relying on relative dimension penalties (EIoU) or reshaping the regression path with angular costs (SIoU). The significant gain in the stricter mAP@0.5:0.95 metric is particularly noteworthy, indicating that FIoU contributes to more precise localization across varying IoU thresholds, which is essential for high-quality inspection.

### 4.5. Ablation Experiment

**Figure 13:**
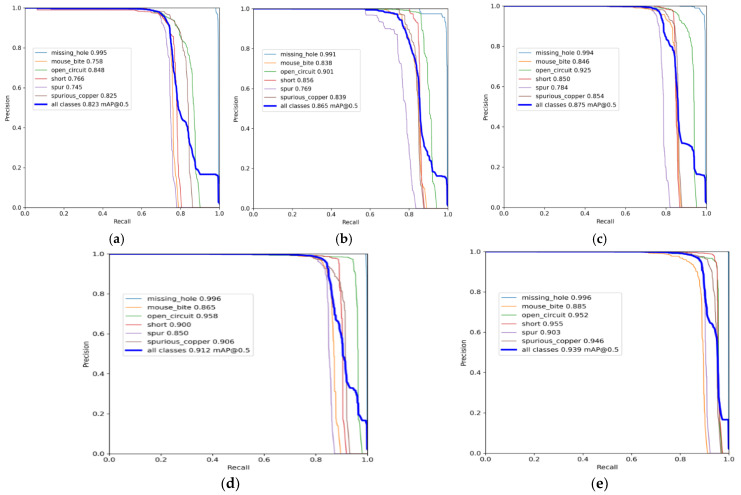
PR curves of experimental results. (**a**) Experiment one. (**b**) Experiment two. (**c**) Experiment three. (**d**) Experiment four. (**e**) Experiment five.

To further validate the effectiveness of the proposed algorithm, this study conducts five ablation experiments based on the original YOLOv5s network using the same dataset, including the original YOLOv5s model, dataset expansion, incorporation of an attention mechanism, addition of a detection head, and replacement of the loss function. A comparative analysis of the ablation results is provided in Table 6 and Table 7, while the Precision–Recall (PR) curves for each experiment are illustrated in Figure 13.

Analysis of the ablation experiments demonstrates the effectiveness of each improvement: Dataset expansion in Experiment 2 increased mAP@0.5 by 4.2%, confirming enhanced generalization and robustness; the attention mechanism in Experiment 3 further improved mAP by 1% and elevated detection accuracy across all defect categories, validating its role in emphasizing target features and suppressing background interference; Experiment 4, with the added small-object detection head, achieved gains of 3.7% in mAP@0.5 and 1.8% in mAP@0.5:0.95, notably improving precision for micro-defects like cracks 1.9%, shorts 5.0%, and burrs 6.6%, proving enhanced utilization of low-level detailed features; finally, replacing CIoU with FIoU in Experiment 5 refined bounding box regression, boosting mAP@0.5 to 93.9% and mAP@0.5:0.95 to 59.6%, with the overall improvement driven significantly by superior detection performance on small targets.

### 4.6. Comparative Experiments with State-of-the-Art Detectors

To clearly define the operational limits of the proposed MFE-YOLO algorithm for practical industrial applications, we analyze its detection performance on defects at critical geometric dimensions. This analysis addresses the minimum detectable feature sizes that are essential for high-precision PCB inspection. The PCB samples in our constructed dataset primarily represent conventional manufacturing processes, with typical design rules including a **minimum conductor clearance of 0.15 mm** and a **minimum drilled hole diameter of 0.2 mm**. These specifications establish the baseline geometric context for our model’s validated performance.

To quantitatively assess the model’s robustness near these manufacturing limits, we conducted a targeted evaluation. We filtered subsets from the test set containing defects whose sizes are at or close to these critical thresholds. Specifically, we identified all “Missing Hole” defect samples with hole diameters **≤ 0.25 mm** and all potential “Short” defect regions where the clearance between conductors is **≤0.18 mm**. The detection performance of MFE-YOLO on these challenging, critically sized subsets is summarized in Table 8.

The results in Table 8 demonstrate that MFE-YOLO maintains high detection accuracy even for defects at the challenging lower bound of typical design rules. For “Missing Hole” defects with diameters down to 0.25 mm, the model achieves an F1-Score of 87.0%. For “Short” defects in regions with clearances as narrow as 0.18 mm—where the thin copper bridge is extremely subtle—the model still attains an F1-Score of 84.4%. This robust performance confirms the efficacy of our improvements, particularly the dedicated small-object detection head and the FIoU loss, in handling the most demanding cases of miniaturization.

**Figure 14:**
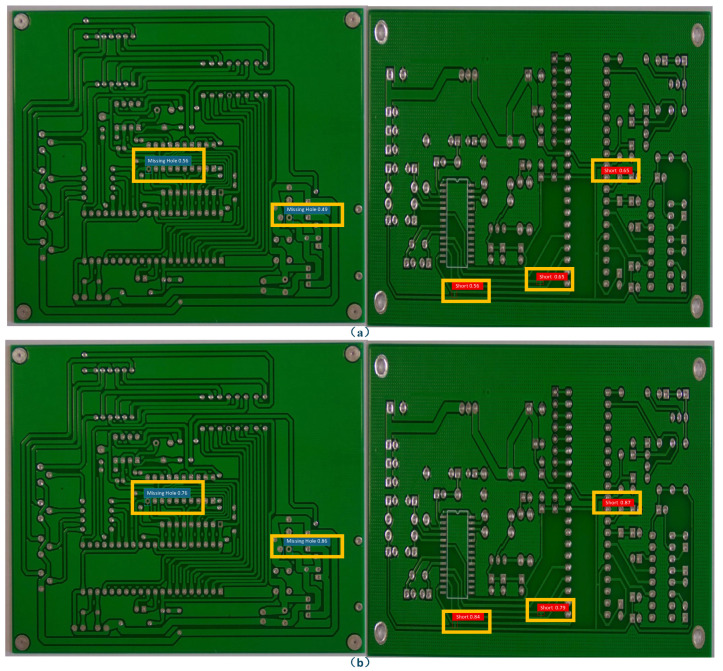
Comparison of Detection Accuracy Improvement for Critical-Size Defects (**a**) Detection Results of the Original Model. (**b**) Detection Results of the Improved MFE-YOLO Model. This figure compares the detection performance of the original YOLOv5 and the improved MFE-YOLO model on critical-size defects.

These quantitative gains are visually substantiated in Figure 14, which provides a side-by-side comparison of detection results for critical-size defects. As shown, the original YOLOv5 model (Figure 14a) produces detection boxes with noticeable localization errors or incomplete coverage for both Missing Hole and Short defects. In contrast, the proposed MFE-YOLO model generates significantly more accurate and complete bounding boxes (highlighted in yellow), aligning closely with the actual defect boundaries. This qualitative improvement directly corresponds to the enhanced precision and recall metrics reported in Table 8, offering concrete visual evidence of how the integrated improvements—the small-object detection head for capturing fine-grained features and the FIoU loss for refined bounding box regression—collectively elevate the model’s capability in pinpointing subtle, miniaturized defects.

### 4.7. Comparison with State-of-the-Art Models

To thoroughly evaluate the performance of the proposed MFE-YOLO model and situate its advancements within the current landscape, we conducted a comprehensive comparison against several prominent and recent object detection architectures. These include the latest iteration in the YOLO series, YOLOv8 [22]; the efficient and scalable EfficientDet-D1 [23]; and the transformer-based end-to-end detector DETR (with a ResNet-50 backbone) [18]. All models were trained and evaluated on our constructed multi-background PCB defect dataset under identical conditions: the same train/val/test split, input image size of 640 × 640, and hardware environment (NVIDIA Quadro M6000). Key performance metrics are reported in Table 9, encompassing detection accuracy (mAP@0.5, mAP@0.5:0.95), model complexity (Parameters, GFLOPs), and inference speed (Frames Per Second, FPS).

As presented in Table 9, the proposed MFE-YOLO achieves the highest detection accuracy among all compared models, attaining 93.9% mAP@0.5 and 59.6% mAP@0.5:0.95. This represents a significant improvement of +2.7% and +1.8% in mAP@0.5 and mAP@0.5:0.95, respectively, over YOLOv8s. This performance gain underscores the collective efficacy of our contributions—the multi-background dataset mitigating data bias, the CBAM enhancing feature discrimination, the dedicated small-object detection head, and the FIoU loss refining localization—particularly for the challenging task of detecting minuscule defects against complex backgrounds.

In terms of model efficiency, EfficientDet-D1 exhibits the lowest computational footprint (6.6M parameters, 11.8 GFLOPs) and the fastest inference speed (158 FPS), benefiting from its compound scaling strategy. However, this efficiency comes at the cost of detection accuracy, which is notably lower than MFE-YOLO, especially on the stricter mAP@0.5:0.95 metric (−4.3%). The transformer-based DETR model, while elegant for its anchor-free and end-to-end design, demonstrates considerably higher complexity (41.0M params, 86.5 GFLOPs) and the slowest inference speed (45 FPS), making it less suitable for scenarios where real-time processing is often required.

MFE-YOLO strikes a compelling balance between accuracy and efficiency. Although it introduces a moderate increase in parameters and GFLOPs compared to YOLOv8s, its inference speed of 128 FPS demonstrates its practical feasibility. The substantial improvement in detection accuracy, especially for critical small defects, justifies this computational cost, as it directly leads to more reliable detection performance.

### 4.8. Analysis of Misclassifications and Model Behavior

To gain deeper insight into the failure modes and limitations of the proposed MFE-YOLO model, we conducted a detailed analysis of misclassified samples from the test set. This analysis aims to identify which defect categories are prone to confusion and to understand the model’s behavior under challenging conditions. The confusion matrix for the six defect categories on the test set, using the final MFE-YOLO model from Experiment Five (Table 6), is presented in Table 10.

A detailed examination of the confusion matrix Table 9 reveals insightful patterns regarding the model’s performance and remaining challenges. The defects Missing Hole and Spurious Copper demonstrate the highest correct classification rates of 98.7% and 97.1%, respectively. Their distinct and localized visual signatures—a clear material absence in a defined area and unintended copper residue—allow the model to learn robust and reliable representations. The primary sources of error are concentrated in two specific confusion pairs. The most significant mutual confusion occurs between Spur and Mouse Bite (4.3% and 1.5%, respectively), both representing geometric imperfections along trace edges. This ambiguity, especially in low-resolution or noisy image regions where differentiating a small protrusion from an indentation is difficult, exemplifies a classic case of aleatoric uncertainty arising from inherent visual similarity. An asymmetric confusion is observed between Short and Open Circuit, where Short circuits are misclassified as Open Circuit 3.1% of the time, more frequently than the reverse (1.4%). This suggests the model may be more sensitive to the strong edge features of a broken trace than to the faint, thin bridge characteristic of a short, potentially misinterpreting an extremely faint connection as a break in continuity.

In particularly ambiguous cases, such as with the Spur defect, which has the lowest individual accuracy (90.3%), errors are distributed across several classes (Mouse Bite, Missing Hole, Short). This indicates that small, localized edge anomalies can activate features associated with multiple defect types. While the integrated CBAM attention mechanism is crucial for focusing on the anomalous region, its efficacy can be limited when the salient distinguishing features are excessively subtle, allowing the final classification to be influenced by secondary, shared characteristics. Furthermore, some confusion, notably between Short and Open Circuit, can be exacerbated by poor localization. An imprecise bounding box that fails to fully encapsulate a thin, short bridge may only capture one side, making the region appear as an open circuit. The proposed FIoU loss function addresses this issue by enhancing bounding box regression stability for small, thin objects, thereby helping to reduce this category of localization-induced misclassification, as evidenced by the improved Average Precision for both classes in Table 7. This fine-grained error analysis confirms that the remaining challenges for MFE-YOLO are semantically meaningful, stemming from genuine visual ambiguities between specific defect pairs. It highlights that future work could focus on developing more discriminative features or loss functions for geometrically similar edge defects and exploring structural reasoning to incorporate connectivity constraints for more reliable distinction between shorts and open circuits.

While a comprehensive per-color quantitative breakdown is not provided in this study, the design of our approach inherently addresses color variability. Firstly, the multi-background dataset explicitly includes PCBs with green, blue, red, black, and white substrates, training the model on a color-diverse prior. Secondly, the integrated CBAM attention mechanism is designed to suppress irrelevant background features (which include color information) and amplify structural and contrast-based defect signatures. The confusion patterns observed in Table 9 (e.g., Spur vs. Mouse Bite) are primarily related to geometric ambiguity rather than background color. The significant overall performance gain on the mixed-color dataset (Table 6, Experiment Five) suggests that the model has learned to generalize across the color variations present in the training data. A dedicated analysis separating performance by substrate color remains an interesting direction for future work to further validate color invariance.

### 4.9. Visualization Experiment

**Figure 15:**
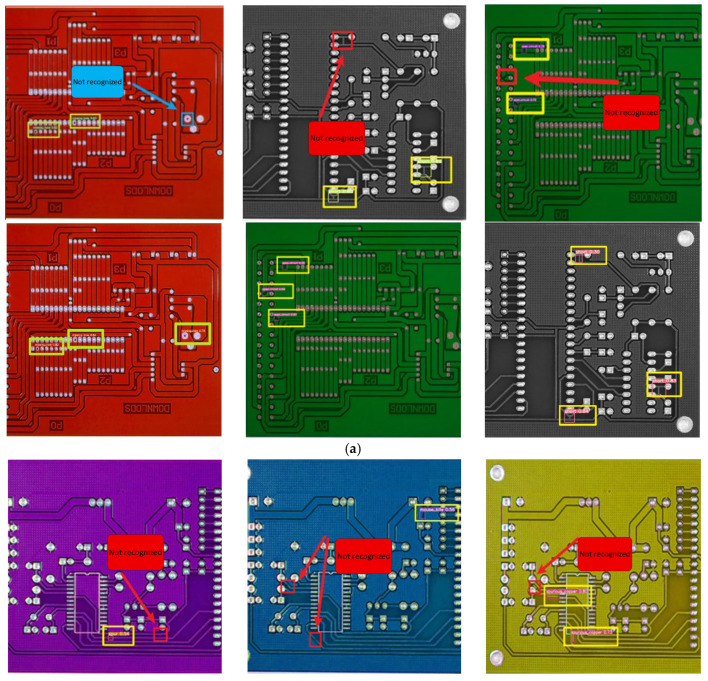

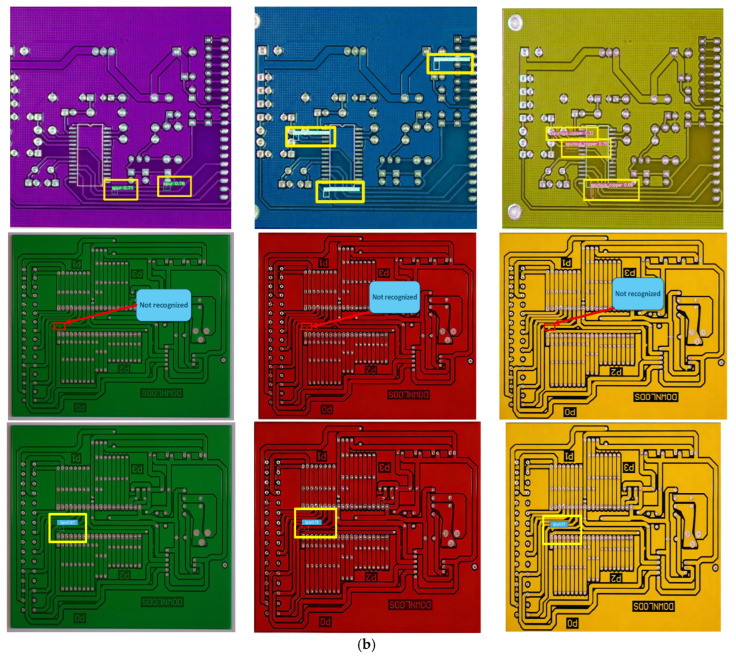
Visual comparison of detection results demonstrating generalization and robustness. (**a**) Generalization test on novel PCB layouts and appearances. (**b**) Robustness test against background color variation on identical PCB layouts.

To further demonstrate the effectiveness of the improved network proposed in this study, visual comparisons were conducted from two perspectives. First, to assess the model’s generalization capability, three additional PCB samples featuring novel circuit layouts and distinct backgrounds were examined. Second, to evaluate its robustness against background variations, three PCB samples with identical circuit layouts but different background colors were tested. The detection results from both the original and improved models are presented in Figure 15.

Figure 15a displays the detection results on PCBs with novel layouts and appearances. When confronted with unseen designs, the original model struggled, resulting in missed detections. In contrast, the improved MFE-YOLO model successfully identified the target defects while maintaining a low false-positive rate. This confirms the model’s strong generalization capability, which is essential for deployment in real-world scenarios with diverse PCB designs.

Figure 15b shows a comparison of detection results on PCBs with identical layouts. It can be observed that the original YOLOv5 model exhibited inconsistent performance: it successfully detected defects on the green background but failed to detect the same defect types on the red and blue backgrounds. Conversely, the improved MFE-YOLO model consistently identified all defects across all three color variations. This indicates that the proposed enhancements—particularly training on the multi-background dataset and the integrated CBAM attention mechanism—effectively suppress color-specific biases, enabling the network to focus on invariant structural defect features and demonstrating excellent color robustness.

In summary, visual evidence from both generalization and controlled scenarios collectively confirms that the proposed MFE-YOLO model significantly improves detection accuracy and effectively reduces the missed detection rate on both unseen PCB layouts and varying background colors. This validates the overall effectiveness and practical utility of our proposed improvements.

## 5. Conclusions

Aiming at the low accuracy of traditional object detection algorithms in detecting PCB defects against complex background colors—especially amid rapid technological advancement, where PCB production processes are becoming increasingly sophisticated, resulting in increasingly smaller defects—most existing algorithms perform relatively poorly on small objects. This paper proposes an improved YOLOv5-based algorithm for PCB defect detection. Experimental results demonstrate that, building upon existing datasets, the improvements made in this study targeting the challenges of PCB defect detection effectively enhance the algorithm’s accuracy in detecting small defects in complex backgrounds. Compared to the original network, the improved algorithm shows a significant increase in detection precision. Although the initial selection of the defect detection model took into account the requirement for detection speed and did not compare its detection speed, considering the subsequent need to meet real-time detection requirements in the PCB industry, future research will incorporate the latest advancements in object detection algorithms and explore network lightweighting techniques to ensure the algorithm meets the demands of being both fast and accurate for PCB defect detection. Furthermore, future work will focus on validating the proposed MFE-YOLO framework on higher-resolution images and more diverse defect scenarios to further enhance its robustness and applicability. The uncertainty quantification capability introduced in this work is expected to be particularly valuable in complex scenarios for prioritizing verification efforts.

## Figures and Tables

**Table 1:** IPC-A-600 Defect Types Classification Table.

Small-Sized Defects	Color Difference Defects	Large-Sized Defects	Round Hole Defects	Other Defect Types
Pinholes	Character defects	**Missing holes**	Misaligned holes	**Open circuits**
Hairline defects	Copper plate color differences	Large-sized over-etching	**Missing holes**	**Short circuits**
Narrow conductor spacing	---	**False copper**	Pinholes	**Notches**
---	---	Missing lines	---	**Burrs**
---	---	Large-sized contaminants	---	Over-etching

**Table 2:** Model Training Parameter Settings.

Parameter Name	Parameter Value
Image_size	640 × 640
Weight_decay	0.0005
Batch_size	8
Learning_rate	0.01
Epochs	300

**Table 3:** Data Augmentation Method Expansion Quantity Table.

Defect Type	Original Quantity/Sheets	Quantity After Data Augmentation/Sheets	Quantity After Cropping/Sheets
Missing hole	280	1120	4060
Mouse bite	271	1084	3822
Open circuit	229	916	3270
Short	204	816	2880
Spur	158	632	2060
Spurious copper	260	1040	3654

**Table 4:** Performance and computational cost of different attention mechanisms.

Model	mAP@0.5 (%)	mAP@0.5:0.95 (%)	Params (M)	GFLOPs	FPS
YOLOv5s	86.5	54.6	7.02	15.8	165
YOLOv5s + SE	86.0	52.7	7.05 (+0.4%)	15.9 (+0.6%)	162 (−1.8%)
YOLOv5s + CA	86.5	54.4	7.03 (+0.1%)	15.9 (+0.6%)	163 (−1.2%)
YOLOv5s + ECA	86.3	53.2	7.02 (+0.0%)	15.8 (+0.0%)	164 (−0.6%)
YOLOv5s + CBAM	87.5	55.1	7.09 (+1.0%)	16.3 (+3.2%)	158 (−4.2%)

**Table 5:** Experimental results of six regression loss functions on the baseline YOLOv5s.

Experiment	mAP@0.5 (%)	mAP@0.5:0.95 (%)
YOLOv5s (CIoU)	86.5	54.6
YOLOv5s + GIoU	85.2	53.7
YOLOv5s + DIoU	86.1	54.4
YOLOv5s + EIoU	87.1	55.4
YOLOv5s + SIoU	87.4	55.7
YOLOv5s + FIoU	88.5	57.9

**Table 6:** Comparison of ablation experiment results.

Experiment	YOLOv5s	Dataset Augmentation	Attention Mechanism	Adding Detection Head	FIoU	mAP@.5(%)	mAP@.5:.95(%)
One	√	---	---	---	---	82.3	53.1
Two	√	√	---	---	---	86.5	54.6
Three	√	√	√	---	---	87.5	55.1
Four	√	√	√	√	---	91.2	56.9
Five	√	√	√	√	√	93.9	59.6

**Table 7:** PCB Comparison of detection accuracy results for six major defect categories.

Experiment	Copper Missing	Notch	Open Circuit	Short Circuit	Burr	False Copper
One	99.1	75.8	84.8	76.6	74.5	82.5
Two	99.1	83.8	90.1	85.6	76.9	83.9
Three	99.4	84.6	92.5	85.0	78.4	85.4
Four	99.6	86.5	95.8	90.0	85.0	90.6
Five	99.6	88.5	95.2	95.5	90.3	94.6

**Table 8:** Detection performance on defects at critical geometric dimensions.

Defect Category	Critical Dimension	Subset Sample Count	Precision	Recall	F1-Score
**Missing Hole**	Hole Diameter ≤ 0.25 mm	127	88.6%	85.4%	87.0%
**Short**	Conductor Clearance ≤ 0.18 mm	89	86.2%	82.7%	84.4%

**Table 9:** Performance comparison between MFE-YOLO and state-of-the-art detectors.

Model	mAP@0.5 (%)	mAP@0.5:0.95 (%)	Params (M)	GFLOPs	FPS
YOLOv8s	91.2	57.8	11.1	28.6	142
EfficientDet-D1	89.7	55.3	6.6	11.8	158
DETR (ResNet-50)	88.4	53.9	41.0	86.5	45
**MFE-YOLO (Ours)**	**93.9**	**59.6**	12.7	32.4	128

FPS is measured on a single Quadro M6000 GPU with a batch size of 1, averaged over three runs.

**Table 10:** Normalized confusion matrix for the six PCB defect types using MFE-YOLO (%).

Actual\Predicted	Missing Hole	Mouse Bite	Open Circuit	Short	Spur	Spurious Copper
Missing Hole	**98.7**	0.3	0.0	0.0	0.5	0.5
Mouse Bite	0.8	**96.2**	1.5	0.0	**1.5**	0.0
Open Circuit	0.0	2.1	**95.2**	**1.4**	0.6	0.7
Short	0.0	0.0	**3.1**	**94.5**	2.4	0.0
Spur	1.2	**4.3**	1.0	1.9	**90.3**	1.3
Spurious Copper	0.9	0.0	0.8	0.0	1.2	**97.1**

## Data Availability

The authors confirm that the data supporting the findings of this study are available within the article.
